# Is Pap Test Awareness Critical to Pap Test Uptake in Women Living in Rural Vietnam?

**DOI:** 10.31557/APJCP.2021.22.3.903

**Published:** 2021-03

**Authors:** Hee Yun Lee, Qingyi Li, Yan Luo, Kun Wang, Sara Hendrix, Jongwook Lee, Sangchul Yoon, Quoc Huy Nguyen Vu

**Affiliations:** 1 *School of Social Work, University of Alabama, Tuscaloosa, AL. *; 2 *Department of Health Sciences, University of Alabama, Tuscaloosa, AL. *; 3 *Department of Applied Economics, University of Minnesota, Minneapolis, MN.*; 4 *Department of Global Health, Graduate School of Public School, Yonsei University, Seoul, South Korea. *; 5 *Hue University of Medicine & Pharmacy, Hue, Vietnam.*

**Keywords:** Pap test, Vietnamese women, Andersen Behavioral Model, cervical cancer screening, awareness

## Abstract

**Introduction:**

Cervical cancer is the second leading cause of cancer death among Vietnamese females. By detecting precancerous cells, Pap test screening plays a critical role in the fight against cervical cancer. The present study aims to investigate health-related factors associated with receipt of Pap test among Vietnamese females living in rural Vietnam, particularly examining the correlation between awareness level of the Pap test and the receiving of Pap test.

**Methods:**

Anderson’s Behavioral Model of Health Services Use was utilized as the present study’s theoretical framework. A self-administrated questionnaire was completed among 193 females residing in Quantri City, Vietnam.

**Results:**

Only 15.5% (N=30) of participants in our sample have had a Pap test. Pap test awareness (OR = 18.38, p<.001) was a strong predictor of Pap test receipt. Participants who had heard about Pap test were 18.38 times more likely to take a Pap test compared to those who had no prior knowledge. Besides the awareness, variables including employment (OR = .18, p<.05), and health insurance coverage (OR = 10.75, p<.05) were significantly associated with Pap test uptake.

**Conclusion:**

Findings from the present study suggests interventions should be provided through public health efforts to enhance awareness of Pap test by aiming at increasing primary prevention of cervical cancer, especially among Vietnamese women living in rural areas, in order to reduce cancer health disparities.

## Introduction

Cervical cancer is the leading cancer and the second most common cause of death in Vietnamese females (Domingo et al., 2008). The incidence rate of cervical cancer in Vietnam affects 4.4 in every 100,000 women (Huynh et al., 2004). Although cervical cancer is a leading cause of death among women in developing countries, it is both preventable and curable (Suba et al., 2001). Globally, cervical cancer screenings have been shown to decrease cervical cancer incidence and mortality among women aged 21 to 65 (US Preventative Task Force, 2018). 

Cervical cancer screening tests, such as the Papanicolaou test (Pap test), are often successful in identifying precancerous cells (American Cancer Society [ACS], 2018). Precancerous cells often go unnoticed due to being asymptomatic, which highlights the necessity of regular cervical cancer screenings (Smith et al., 2017). In Vietnam, preventative measures began in 1996 with a Memoranda of Understanding to begin Pap screening services for women, which sought to establish sustainable, cost-effective cervical cancer prevention services for women (Suba et al., 2001; Suba et al., 2006). To date, the cost of a Pap test and screening services remain inexpensive; however, the Pap test receipt in Vietnam still remains low (Domingo et al., 2008; Suba et al., 2001, 2006; Tran et al., 2018). 

A number of sociodemographic factors are correlated with the uptake of Pap test screening including age, marital status, education, and income (Lee-Lin et al., 2007; Lee et al., 2015; Randolph et al., 2002; Akinlotan et al., 2018). Additionally, health insurance coverage has been associated with being the most important factor predicting Pap test screening (Do, 2015; Nguyen-Truong et al. , 2012; Luque et al., 2018). The knowledge of human papillomavirus (HPV), Pap test screening, and cervical cancers are also factors associate with Pap test screening ( Thompson et al., 2018; Ashtarian et al., 2017). Research has demonstrated a focus on cost-effectiveness analysis, quality management and sexual risk factors for uptake of the Pap test, and has limited findings in the role of health insurance or Pap test awareness (Suba et al., 2001; Suba et al., 2005; Suba et al., 2006; Kim, Kobus et al., 2008; Tran et al., 2015). 

In this study, Andersen’s Behavioral Model of Health Service Utilization was used as framework to investigate factors related to Vietnamese’s uptake of Pap test. Andersen’s Behavioral Model of Health Service Utilization was a widely used model to explain factors associated with the utilization of health services (Andersen, 1995). Determinants of health service utilization could be categorized into three dimensions: predisposing factors, enabling factors and need factors (Andersen, 1995). In this study, predisposing factors are demographic information that cannot or can hardly be changed, such as age, marital status, employment status, and education level; enabling factors are those highly mutable factors, such as health insurance coverage, cervical cancer literacy, usual medical care place, and Pap test awareness; need factors include perceived and actual needs, such as family cancer history, personal cancer history, and self-rated health status. 

To our knowledge, the present study is the first in exploring the role of Pap test awareness in uptake of Pap test screening among Vietnamese women. Therefore, it is imperative to investigate the relationship between health-related behavior factors and Pap test receipts in Vietnam. Considering the research gap on the factors of Pap test uptake and the association between Pap test awareness and Pap test receipt among Vietnamese women, the purpose of this study was to examine health-related factors associated with receipt of Pap test among Vietnamese women, particularly the correlation between awareness level of the Pap test and the receipt. The study addresses the following research questions:

- What is the level of Pap test receipt among the population of Vietnamese women?

- What are the risk factors associated with the receipt of Pap test?

- What is the relationship between Pap test awareness and the screening receipt after relevant variables are controlled?

## Materials and Methods

*Research Design and Data Collection*

This study was part of a larger research program on the investigation of the prevention behaviors of different cancers, such as breast cancer, colorectal cancer, and cervical cancer in Vietnam. Collaborated with a local organization, a convenient sample of Vietnamese aged 18 or older were collected in rural areas in Quang Tri Province in 2017. A cross-sectional research design was adopted in this study. The self-administered questionnaire took 20-30 to complete. Before starting the questionnaire, informed consent was obtained from participants. A total of 193 Vietnamese women were recruited, and one participant aged over 65-year old was excluded from our study based on the population recommendation of U.S. Preventive Services Task Force. Thus, 192 participants were used for analysis. This study was approved by the Institutional Research Board (IRB) at the authors’ university.

*Instrument*

Dependent variable. The outcome variable of this study was the receipt of Pap test, which was measured by a dichotomous value (0= no, 1= yes).

Independent variables. We utilized Andersen Behavioral Model to categorize independent variables which were divided into predisposing, enabling, and need variables. 

Predisposing variables. Age, marital status, employment, and education level were selected as predisposing variables. Age was a continuous variable in our study. Dichotomous values were used for marital status (0= others, 1= married or partnered), employment (0= no, 1= yes), and education (0= <Bachelor’s degree, 1= ≥Bachelor’s degree).

Enabling variables. Health insurance coverage, cervical cancer literacy, having usual medical care place, Pap test awareness were entered to enabling variable category. Yes/no questions (0= no, 1= yes) were conducted to measure whether having health insurance coverage, having usual medical care place, and having Pap test awareness. Cervical cancer literacy was measured by a 5-item scale. Each of the items was a true/false question (0= false, 1=true), and the number of correct answers was the cervical cancer literacy score, which was treated as a continuous variable (0-5). 

Need variables. Need variables included family cancer history, personal cancer history, and self-rate health status. Personal cancer history and family cancer history were dichotomized (0= no, 1= yes). A 5-point Likert scale was used to measure self-rated health status and the self-rated health score was regarded as a continuous variable (0-4) in our study. 

*Data Analysis*

Univariate analysis was used to examine sociodemographic characteristics (age, marital status, employment, and education level), health accessibility (health insurance coverage, and having usual medical care place), health history information (family cancer history, personal cancer history, and self-rated health score) and health literacy information (Pap test awareness, and cervical cancer literacy). Bivariate analysis was conducted to test the relationship between these variables and the receipt of Pap test. Chi-square test was used for categorical variables, such as marital status, employment, education level, health insurance coverage, having usual medical care place, family cancer history, personal cancer history, and Pap test awareness. T-test was performed for continuous variables, such as age, self-rated health score and cervical cancer literacy. Logistic regression analysis predicting Pap test receipt was conducted with predisposing, enabling, and need variables. All analysis of this study was conducted using SPSS 25.0.

## Results

*Participant Characteristics*

A total of 192 Vietnamese women were analyzed in our study. Nearly half (49.5%) participants were aged 21-39 years, and the other half (50.5%) were aged 40-59 years. The majority of the women (91.7%) were married or partnered and employed (89.1%). Only 17.2% women held bachelors’ degree or higher. With regard to health access, 81.8% of our participants were covered by health insurance, 65.1% had a usual medical care place. For health information, about 5.2% had personal cancer history, 25.5% had family cancer history and only 36.5% had ever heard about Pap test.

As Table 1 indicates, among those who received Pap test, significant differences were found in education (*χ*^2^ = 6.512, ρ< .05), health insurance coverage (*χ*^2^ = 5.293, ρ<0.05), and Pap test awareness (*χ*^2^ = 33.724, ρ<0.001). There were no significant differences in age, marital status, employment, having usual medical care place, personal cancer history and family cancer history. In addition, no significant differences were found in self-rated health score and cervical cancer literacy.

*Receipt of Pap Test by age and Time Frame*

As shown in Table 2, 15.5% (N=30) participants in our samples had a Pap test in their life time, among which 17.9% (N=17) were aged 21-39 years and 13.4% (N=13) were aged 40-59 years. About 13% of participants aged from 21-39 years (N=12) and 3% of participants in 40-59 years group (N=8) had Pap test within 3 years. Moreover, only 4% of all participants had Pap test within in a year in the present study. Approximately 5% of participants in 21-39 years (N=5) and 40-59 yeas (N=5) took Pap test more than 3 years ago, respectively. 

*Logistic Regression Analysis on Pap Test Receipt*

Among the predisposing variables, only employed (OR = .184, p<0.05) were significantly associated with Pap test receipt. Participant who were employed showed significant lower likelihood of uptake Pap test in the present study. As for the enabling factors, health insurance coverage (OR = 10.749, p<0.05) was significant. Those who had health insurance coverage were 10.75 times that of those who do not have insurance to take Pap tests. Pap test awareness (OR = 18.377, p<0.001) was a strong significant predictor. Participants who were aware of Pap test were 18.38 times that of those who were not aware of it to take Pap test. All of the need variables were not significantly associated with receipt of Pap tests. 

**Table 1:** Sociodemographic Characteristics of the Sample (N=192)

Characteristic	n	%	Received Pap Test		
			n	%	
Age					
(M=40.70, SD=5.357)					
21-39 years	95	49.5	17	17.9	0.735
40-59 years	97	50.5	13	13.4	
Marital Status					
Married or partnered	176	91.7	27	15.3	0.129
Other	16	8.3	3	18.8	
Employed					
Yes	171	89.1	26	15.2	0.21
No	21	10.9	4	19	
Education Level					
<Bachelor’s degree	159	82.8	20	12.6	6.512*
Bachelor’s degree	33	17.2	10	30.3	
Health insurance coverage					
Yes	157	81.8	29	18.5	5.293*
No	35	18.2	1	2.9	
Having usual medical care place					
Yes	125	65.1	21	16.8	
No	67	34.9	9	13.4	0.375
Family cancer history					
Yes	49	25.5	9	18.4	0.375
No	143	74.5	21	14.7	
Personal cancer history					
Yes	10	5.2	1	10	0.253
No	182	94.8	29	15.9	
Pap test awareness					
Yes	70	36.5	25	35.7	33.724**
No	122	63.5	5	4.1	
	M	SD	Received Pap Test		
			M	SD	t
Age	40.7	5.357	39.87	4.688	0.931
Self-rated health score	2.06	0.598	2.27	0.64	-1.978
Cervical cancer literacy	2.73	1.601	2.77	1.073	-0.162

* p < 0.05, ** p < 0.001

**Table 2:** Receipt of Pap Test by Age and Time Frame (N=192)

Age									
	21-39 (n=92)			40-59 (n=82)			Total		
Time	n	%	Cum.	n	%	Cum	n	%	Cum
Ever had	17	17.9		13	13.4		30	15.5	
1 year	5	5.3	5.3	3	3.1	3.1	8	4.1	4.1
1, 2 years	5	5.3	10.6	3	3.1	6.2	8	4.1	8.2
2, 3 years	2	2.1	12.7	2	2.1	8.3	4	2.1	10.3
3 years	5	5.3	17.9	5	5.2	13.4	10	5.2	15.5

**Table 3:** Logistic Regression Analysis on Pap Test Uptake (N=192)

Factors	Variables	Received Pap test			
		B	OR	95% CI	
Predisposing	Age (29~54)	-0.085	0.919	[0.831, 1.015]	
	Marital status	-1.101	0.332	[0.063, 1.745]	
	(Ref= not married or partnered)				
	Employed (Ref= not employed)	-1.691	.184*	[0.034, 0.994]	
	Education level	0.425	1.529	[0.483, 4.840]	
	(Ref= less than bachelor’s degree)				
Enabling	Health insurance coverage (Ref= no)	2.375	10.749*	[1.189, 97.187]	
	Cervical cancer literacy (0~5)	-0.124	0.884	[0.619, 1.262]	
	Having usual medical care place (Ref= no)	-0.186	0.83	[0.284, 2.429]	
	Pap test awareness (Ref= no)	2.911	18.377**	[5.409, 62.439]	
Need	Family cancer history (Ref= no)	-0.2	0.818	[0.281, 2.381]	
	Personal cancer history (Ref= no)	-0.94	0.391	[0.036, 4.211]	
	Self-rate health score (0~4)	0.4	1.491	[0.684, 3.251]	
Constant		0.011	1.011		
Pseudo *R*^2^		0.394			
*χ* ^2^		9.347 (*p=0.314*)			
-2 Log likelihood		116.678			

Pseudo *R*^2^ is Nagelkerke’s *R*^2^; B, coefficient; OR, odds ratio; *p<0.05, **p<0.001

## Discussion

The present study investigated the level of Pap test receipts and health-related factors associated with Pap test uptake among Vietnamese females guided by Andersen’s Behavioral Model of Health Services. This study provided the first empirical evidence about the low level of Pap test receipt among females in Vietnam. Approximately 16% of participants have had a Pap test in their lifetime and only 4% of participants received a Pap test within a year, in the present study. Vietnamese female participants who were employed showed a significantly lower level of Pap test receipt, which was consistent with the literature (Maxwell et al., 2000). This finding revealed that demographic characteristics are also important aspects to investigate and understand Vietnamese females who have not gotten the Pap test. One plausible explanation is that Vietnamese females who are employed tend to be occupied by their work and have less opportunities to receive cancer screenings than those who were unemployed. This result indicated that cancer prevention programs should target both employed and unemployed females to increase Pap test receipt. 

Among enabling factors, participants who had health insurance revealed 10 times higher likelihood to receive a Pap test than those who were not insured among Vietnamese females in the present study, which has mirrored previous studies (Do, 2015; Yi et al., 2013). This result indicates essential implications in increasing the coverage of health insurance, thus improving cancer preventive behaviors and reducing health disparities in Vietnam. 

More importantly, Vietnamese female participants who had heard of Pap tests or were aware of the Pap test was the strongest factor associated with the receipt of Pap tests which is in line with prior studies (Lam et al., 2003; Nguyen et al., 2012). Vietnamese females who have heard of Pap test were 18 times more likely to receive a Pap test compared to those who have never heard of one. This finding indicates that Pap test awareness is crucial for improving the Pap test receipt among females in Vietnam.

*Implications for Nursing Practice and Research*

Despite the limitations of the present study, a number of significant implications for nursing and health care practice and policy surfaced. The findings highlighted the importance of studying Vietnamese females, as well as studying their cultural and contextual factors related to health and cancer preventive behaviors. The present study identified two significant modifiable barriers to Pap test receipt in Vietnam, (1) the awareness of Pap test and (2) owning health insurance, which have fundamental implications for health practice and policy. 

First, a lack of awareness or knowledge of the Pap test is the most critical barrier of getting a Pap test in Vietnam (Lam et al., 2003; Nguyen et al., 2012). Community-based educational programs could be essential to increase the Pap test receipt. Health message delivery through media campaigns and social network sites (e.g., Facebook or twitters) offer an innovative future intervention to increase the awareness of cervical cancer and the Pap test in Vietnamese communities. More importantly, future studies and practice should also pay attention on how to transfer the awareness of cervical cancer screening to an actual Pap test receipt in Vietnam (Fang et al. , 2011). Second, having health insurance could promote Pap test receipts among Vietnamese females. The present findings suggest that expanding health insurance coverage and increasing the financial accessibility of the Pap test is essential for Vietnamese females (Do, 2015). Finally, the findings presented in the current study provide the opportunity for nurses and health care professionals to explore and implement strategies to close the health disparity gap among adult women living in rural Vietnam. 

*Limitations*

Although the present study provides valuable insight into health-related factors associated with receiving Pap tests among Vietnamese females, several limitations should be considered. First, the present study was a cross-sectional study which did not reveal causal relationships between factors and Pap test receipt. Next, the present study employed a small sample size of females in Vietnam. Therefore, the findings of this study need to be cautious when generalizing to Vietnamese females in Vietnam. However, the small sample size might not be a significant limitation due to the present results being consistent with previous studies. Future longitudinal studies would be needed to examine the causal relationships between health-related factors and the Pap test receipt among Vietnamese females and other Asian females. 

In Conclusion, cervical cancer is the most common cancer among females in Vietnam which should be addressed by public health policies. Pub1ic health-related policies should advocate the screening for early detection, develop new techniques for cervical cancer diagnosis, and form a national medical network for cervical cancer prevention in Vietnam (Domingo et al., 2008). Literature supports that many women may be ignoring the importance of Pap testing, which additionally emphasizes the need for educational programs (Hsu et al., 2011). This study provides a step toward a better understanding of the barriers to Pap test receipts in Vietnam. The present study highlights the significant role of Pap test awareness. It is necessary and urgent for public health efforts to be focused on increasing the primary prevention of cervical cancer. This can be achieved by focusing on increasing the awareness of Pap tests in communities in order to reduce cervical cancer screening disparities in Vietnam (Domingo et al., 2008; Fang et al., 2011). 

## Author Contribution Statement

None.
